# O Uso de um Ventilador Mecânico Portátil na Ressuscitação Cardiopulmonar é Viável, Melhora os Parâmetros Respiratórios e Previne a Redução da Complacência Pulmonar Dinâmica

**DOI:** 10.36660/abc.20220564

**Published:** 2023-07-19

**Authors:** Manoel Ângelo Gomes Palácio, Edison Ferreira de Paiva, Gustavo Bernardes de Figueiredo Oliveira, Luciano César Pontes de Azevedo, Bruno Gregnanin Pedron, Elizabete Silva dos Santos, Ari Timerman

**Affiliations:** 1 Instituto Dante Pazzanese de Cardiologia São Paulo SP Brasil Instituto Dante Pazzanese de Cardiologia, São Paulo, SP – Brasil; 2 Hospital Sírio-Libanês São Paulo SP Brasil Hospital Sírio-Libanês, São Paulo, SP – Brasil; 3 Universidade de São Paulo São Paulo SP Brasil Universidade de São Paulo, São Paulo, SP – Brasil

**Keywords:** Hiperventilação, Lesão Pulmonar, Ressuscitação Cardiopulmonar, Ventiladores Mecânicos, Complacência Pulmonar

## Abstract

**Fundamentos:**

Para ventilação prática e protetora durante a ressuscitação cardiopulmonar (RCP), desenvolveu-se um ventilador mecânico (VLP2000E) de 150 gramas que limita o pico de pressão inspiratória (PPI) durante ventilação e compressões torácicas simultâneas.

**Objetivos:**

Avaliar a viabilidade da ventilação com VLP2000E durante RCP e comparar os parâmetros monitorados versus ventilação com bolsa-válvula.

**Métodos:**

Estudo experimental randomizado com 10 porcos intubados por grupo. Após sete minutos de fibrilação ventricular, iniciaram-se ciclos de RCP de 2 minutos. Todos os animais foram ventilados com VLP2000E após o retorno da circulação espontânea (RCE).

**Resultados:**

Os grupos bolsa-válvula e VLP2000E apresentaram taxa de RCE (60% vs. 50%, respectivamente) e saturação arterial de oxigênio similares na maioria dos ciclos de RCP, volume corrente basal diferente [0,764 (0,068) vs. 0,591 (0,123) L, p = 0,0309, respectivamente] e, em 14 ciclos, diferentes PPI [52 (9) vs. 39 (5) cm H_2_O, respectivamente], volume corrente [0,635 (0,172) vs. 0,306 (0,129) L], ETCO_2_ [14 (8) vs. 27 (9) mm Hg], e pico de fluxo inspiratório [0,878 (0,234) vs. 0,533 (0,105) L/s], todos p < 0,0001. A complacência pulmonar dinâmica (≥ 0,025 L/cm H_2_O) diminuiu após o RCE no grupo bolsa-válvula, mas se manteve no grupo VLP2000E [ 0,019 (0,006) vs. 0,024 (0,008) L/cm H_2_O, p = 0,0003].

**Conclusões:**

Ventilação com VLP2000E durante RCP é viável e equivalente a ventilação com bolsa-válvula quanto à taxa de RCE e saturação arterial de oxigênio. Esse ventilador produz melhores parâmetros respiratórios, com pressão das vias aéreas e volume corrente menores. Ventilação com VLP2000E também previne a redução significante da complacência pulmonar dinâmica observada após ventilação com bolsa-válvula. Seria interessante realizar mais estudos pré-clínicos para confirmar esses resultados.

## Introdução

A ressuscitação cardiopulmonar (RCP) de alta qualidade com adequada ventilação é atualmente priorizada, porém a hiperventilação continua sendo um problema, mesmo quando a RCP é realizada por equipes bem treinadas.^1 , 2^ Assim, soluções são necessárias para se guiar, como o uso de métodos de monitoramento da ventilação.^2^ Além de causar efeitos hemodinâmicos deletérios, altas frequências respiratórias e volumes correntes elevados durante RCP podem lesar os pulmões.^1 , 2^ Em pacientes críticos sob ventilação mecânica, recomendam-se cuidados para evitar barotrauma, volutrauma e atelectrauma.^3^ Dessa maneira, tem-se observado diminuição de morbidade e mortalidade nos casos de lesão pulmonar aguda e síndrome da angústia respiratória com reduções no volume corrente e na pressão das vias aéreas.^3^

Objetivando uma ventilação prática e protetora durante a RCP, desenvolveu-se um ventilador mecânico portátil que limita o pico de pressão inspiratória (PPI), o VLP2000E (Vent-Logos Ltda., Vitória, ES, Brasil). O estudo testou o VLP2000E em um modelo animal de parada cardíaca súbita. Os objetivos foram avaliar a viabilidade de ventilação com VLP2000E durante RCP e comparar os parâmetros monitorados versus ventilação com bolsa-válvula.

## Métodos

Este foi um estudo experimental randomizado em um modelo de parada cardíaca súbita e RCP utilizando porcos, delineado para simular um caso de parada cardíaca em adulto fora do hospital. Vinte porcos foram randomizados 1:1 para ventilação com bolsa-válvula ou ventilação com VLP2000E. O estudo foi aprovado pela Comissão de Ética no Uso de Animais (protocolo número CEUA 2015-13 IEP-HSL).

### Detalhes do VLP2000E

O VLP2000E é um ventilador mecânico pneumático, com ciclagem por tempo, limitado por pressão, pesando 150 gramas. O ventilador funciona automaticamente quando conectado a um cilindro portátil de oxigênio, com fácil ajuste da frequência respiratória. Além disso, a ventilação também pode ser manualmente acionada apertando-se um botão. O ventilador tem mecanismos que permitem respiração espontânea, e uma válvula especial que limita o PPI mesmo durante ventilação e compressões torácicas simultâneas.

### Preparo

Porcos fêmeas da raça Landrace, pesando aproximadamente 33 kg, ficaram em jejum por 12 horas com livre acesso à água antes do procedimento. Após anestesia intramuscular [5 mg/kg de cloridrato de cetamina (Ketalar 50 mg/mL, Pfizer); 0,5 mg/kg de midazolam (Dormonid 5 mg/mL, Roche)], 12,5 mg/kg de tiopental (Thiopentax 20 mg/mL, Cristália) foi infundido por uma veia da orelha, e o animal foi intubado com um tubo endotraqueal número sete com manguito, que foi inflado para evitar vazamentos. A anestesia endovenosa contínua foi mantida com midazolam 1,5 mg/kg/h e 0,015 mg/kg/h de citrato de fentanila (Fentanil 0,05 mg/mL, Janssen) e doses ilimitadas de 4 mL em bolos, além de tiopental 0,6-6 mg/kg/h.

Um ventilador mecânico convencional Dräger Evita XL (Drägerwerk AG & Co. KGaA, Lübeck, Alemanha) foi utilizado somente durante o preparo, ajustado em pressão positiva intermitente, volume corrente de 10 mL/kg, pressão positiva expiratória final (PEEP) de 5 cm H_2_O, e fração de oxigênio inspirado (F_IO2_) de 50%; a frequência respiratória foi ajustada para manter o dióxido de carbono expirado final (ETCO_2_) entre 40 e 45 mm Hg.

As veias jugulares e as artérias femorais dos animais foram puncionadas e canuladas com introdutores hemostáticos de 8 French (F) e 6 F, respectivamente. A veia jugular direita foi usada para a infusão de anestesia geral e todos os medicamentos. Um cateter Swan-Ganz de 7,5 F com débito cardíaco contínuo foi posicionado na artéria pulmonar via veia jugular esquerda, e um cateter *pigtail* de 6 F foi posicionado na raiz da aorta via artéria femoral direita.

### Monitoramento, parâmetros medidos e coleta de sangue

Os seguintes equipamentos foram utilizados: monitor Dräger Infinity Delta XL (Dräger Medical Systems Inc., Telford, PA, USA); monitor NICO ETCO_2_ (Koninklijke Philips Electronics NV, Amsterdã, Holanda); monitor de débito cardíaco Vigilance II (Edwards Lifesciences LLC, Irvine, CA, EUA); monitor das vias aéreas MVA1000 (Neurony Ltda., Vitória, ES, Brasil); sistema de aquisição de dados Biopac MP100 e programa AcqKnowledge (Biopac Systems Inc., Goleta, CA, EUA); e analisador de gasometria Radiometer ABL735 (Radiometer Medical ApS, Brønshøj, Dinamarca).

O sistema Biopac gravou os seguintes parâmetros a 250 amostras por segundo: eletrocardiograma (eletrodos de superfície Biopac); pressão aórtica (transdutor de pressão Biopac conectado ao cateter *pigtail* ); pressão atrial direita (transdutor Biopac conectado à porta atrial do Swan-Ganz); pressão de perfusão coronária (PPC) (diferença entre pressão aórtica e atrial direita); ETCO_2_ (saída analógica do NICO conectada ao Biopac); débito cardíaco (saída analógica do Vigilance); pressão e fluxo das vias aéreas (saídas analógicas do MVA1000). Calcularam-se o volume corrente (integral da curva de fluxo na inspiração) e uma complacência pulmonar dinâmica simplificada (razão do volume corrente pelo PPI). A temperatura central também foi monitorada.

Coletaram-se amostras de sangue da artéria femoral esquerda (ou da aorta durante RCP) 30 minutos antes da parada cardíaca (basal), nos ciclos de RCP 2, 4, 7, 9, 12 e 14, e após o retorno da circulação espontânea (RCE) aos 10, 30, 60, 90 e 120 minutos. Parâmetros da gasometria, respiratórios e hemodinâmicos (exceto o débito cardíaco que usualmente não é mensurável na RCP) foram medidos nesses ciclos e momentos.

### Sistematização das medidas

Em todos esses ciclos e momentos, selecionaram-se três respirações consecutivas usando o programa AcqKnowledge para a medida de cada parâmetro e obtenção da média final dos parâmetros respiratórios e hemodinâmicos. Os seguintes intervalos foram definidos para as medidas: ciclo respiratório total, intervalo entre dois complexos QRS ou duas compressões torácicas, e intervalo da inspiração, obtendo-se as frequências respiratória, cardíaca, e de compressão torácica, o PPI, o pico de fluxo inspiratório (PFI), o volume corrente e a complacência pulmonar dinâmica. No intervalo do ciclo respiratório total, mensuraram-se o ETCO_2_, a pressão aórtica, pressão atrial direita, PPC, e o débito cardíaco.

### Experimento

A fibrilação ventricular foi induzida pela aplicação direta de corrente de uma bateria de 9 V no ventrículo direito por um segundo, via eletrodo de marca-passo transvenoso posicionado temporariamente pela veia jugular direita. Após sete minutos de inatividade para simular o tempo de resposta dos serviços médicos de emergência, iniciaram-se ciclos de RCP de 2 minutos (100 compressões torácicas manuais por minuto, com aproximadamente 5 cm de profundidade e 10 ventilações por minuto). Dez porcos foram ventilados com bolsa-válvula para adulto com reservatório e influxo de 10 L/min de oxigênio, e outros 10 porcos foram ventilados com VLP2000E e F_IO2_ de 100%. Em ambos os grupos não se aplicou PEEP, e o oxigênio proveio de um cilindro portátil. Guiado por um metrônomo, para se evitar viés interpessoal, um mesmo membro da equipe comprimiu o tórax a 100 compressões por minuto; um outro membro aplicou 10 ventilações por minuto com bolsa-válvula, aplicando cada ventilação em aproximadamente dois segundos acompanhando quatro batidas do metrônomo, e reiniciando a ventilação na décima batida. O VLP2000E foi ajustado para 10 ventilações por minuto. Após o primeiro ciclo de RCP, tentou-se a desfibrilação com 150 J e se retomou a RCP imediatamente. O ritmo cardíaco foi verificado após cada ciclo e, conforme a indicação, seguiram-se outras tentativas com 150 J ou retomada da RCP. Nos ciclos 2, 4, 7, 9, 12 e 14, administrou-se 0,02 mg/kg de adrenalina (Epinefrina 1 mg/mL, Cristália). Nos ciclos 3 e 5, administraram-se 5,0 mg/kg e 2,5 mg/kg de amiodarona (Ancoron 50 mg/mL, Libbs), respectivamente, nos casos de fibrilação ventricular persistente. A RCP foi mantida por até 30 minutos ou até o RCE e, em casos de RCE, todos os animais foram colocados no VLP2000E por duas horas antes da eutanásia com anestésicos e 10 mL de cloreto de potássio. RCE sustentado era indicado por ritmo cardíaco adequado e pressão sistólica acima de 50 mm Hg por mais de 10 minutos e, se parada cardíaca espontânea ocorresse após, o experimento terminava; mas se ocorresse antes de 10 minutos, reiniciava-se a ressuscitação até completar 30 minutos de RCP ou até o RCE. Após cada episódio de RCE em ambos os grupos, aplicavam-se cinco ventilações consecutivas vigorosas com bolsa-válvula para expandir os pulmões igualmente nos dois grupos.

### Análise estatística

Partindo da premissa de que a ressuscitação não seria prejudicada por ventilação mecânica, o tamanho da amostra foi calculado assumindo-se uma taxa de RCE de 83%. Caso contrário, se apenas 25% de RCE fosse alcançado no grupo submetido à ventilação mecânica, seriam necessários 10 animais por grupo para poder estatístico de 80% e alfa bicaudal de 5%.

Utilizou-se o programa de estatística BioStat (AnalystSoft, Inc.) para analisar os resultados com os seguintes testes: qui-quadrado ou teste exato de Fisher, ANOVA de dois fatores (ciclo de RCP ou momento, grupo) e compações múltiplas de Bonferroni. Os gráficos foram gerados no programa Number Cruncher Statistical System (NCSS, LLC). Os dados foram apresentados como média (desvio padrão) [média (DP)], e consideraram-se as diferenças significantes com p < 0,05.

## Resultados

**Figura 1:**
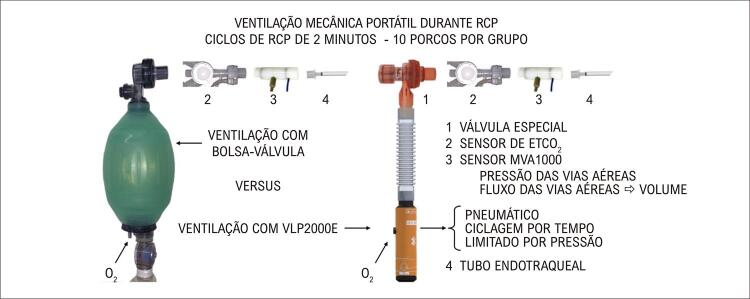
– A bolsa-válvula e o VLP2000E, e o posicionamento dos sensores respiratórios; RCP: ressuscitação cardiopulmonar; ETCO2: dióxido de carbono expirado final.

A preparação dos animais, cujo peso médio foi 33 (2) kg, demorou aproximadamente 90 minutos. Durante o experimento, a temperatura central média foi 37,3 (1,3) °C. Após a preparação e ainda sob ventilação mecânica convencional, os valores dos parâmetros monitorados são apresentados na Tabela 1 . A Figura 1 ilustra a bolsa-válvula e o VLP2000E com acessórios usados no experimento, além de mostrar esquematicamente a posição dos sensores respiratórios.

**Tabela 1:** – Parâmetros medidos durante ventilação mecânica convencional antes da parada cardíaca, segundo o dispositivo de ventilação usado na ressuscitação cardiopulmonar

PARÂMETRO	BOLSA-VÁLVULA	VLP2000E	p

	10 porcos	10 porcos	
P_aCO2_ (mm Hg)	44 (4)	43 (4)	0,8631
P_aO2_ (mm Hg)	213 (19)	221 (16)	0,7822
S_aO2_ (%)	99,9 (0,2)	99,9 (0,2)	0,9964
ETCO_2_ (mm Hg)	43 (5)	42 (5)	0,7274
Frequência respiratória (rpm)	16 (4)	16 (4)	0,6636
Frequência cardíaca (bpm)	74 (15)	62 (13)	0,2277
Débito cardíaco (L/min)	2,7 (0,7)	2,5 (0,6)	0,5523
Pressão aórtica (mm Hg)	93 (21)	96 (17)	0,8107
Pressão atrial direita (mm Hg)	13 (2)	12 (2)	0,7635

Valores apresentados como média (desvio padrão); a: sangue arterial; P_CO2_: pressão parcial de dióxido de carbono; P_O2_: pressão parcial de oxigênio; S_O2_: saturação de oxigênio; ETCO_2_: dióxido de carbono expirado final.

### RCE e sobrevida em duas horas

Taxas similares (p = 1,0000) de RCE foram alcançadas no grupo bolsa-válvula (60%; três porcos após dois ciclos de RCP, três porcos após três ciclos) e no grupo VLP2000E (50%; dois porcos após dois ciclos de RCP, três porcos após três ciclos). Após 30 minutos de observação, três animais do grupo bolsa-válvula sofreram parada cardíaca espontânea. Às 2 horas de observação, três animais do grupo bolsa-válvula e cinco animais do grupo VLP2000E estavam vivos (p = 0,6499).

### Parâmetros monitorados durante a RCP

Alguns parâmetros não diferiram entre os grupos bolsa-válvula e VLP2000E em todos os ciclos de RCP, incluindo a frequência de compressão torácica [98 (2) vs. 99 (2) cpm, p = 0,8820], frequência respiratória [11 (1) vs. 11 (1) rpm, p = 0,4477], e PPC, por exemplo, no ciclo 1 [30 (17) vs. 35 (16) mm Hg, p = 0,6207], respectivamente. Os parâmetros da gasometria não diferiram entre os grupos na maioria dos ciclos de RCP, exceto por, inicialmente, hipercapnia relativa no grupo VLP2000E ( Tabela 2 ).

**Tabela 2:** – Gasometria arterial analisada durante a ressuscitação cardiopulmonar (RCP), segundo o dispositivo de ventilação usado. Inicialmente com 10 porcos por grupo, do quarto ciclo de RCP ao 14º ciclo, o grupo bolsa-válvula incluiu quatro porcos e o grupo VLP2000E incluiu cinco porcos

RCP	GRUPO	P_aCO2_	p	P_aO2_	p	S_aO2_	p
		mm Hg		mm Hg		%	
Ciclo 2	Bolsa-válvula	27 (8)	0,0056	149 (63)	0,1040	97 (3)	0,0912
	VLP2000E	52 (13)		103 (90)		86 (13)	
Ciclo 4	Bolsa-válvula	35 (12)	0,0337	109 (99)	0,3025	79 (24)	0,3232
	VLP2000E	64 (19)		65 (20)		69 (22)	
Ciclo 7	Bolsa-válvula	29 (2)	0,0111	83 (24)	0,6193	90 (7)	0,0105
	VLP2000E	72 (20)		56 (13)		58 (21)	
Ciclo 9	Bolsa-válvula	94 (92)	0,6098	41 (44)	0,8552	45 (59)	0,8714
	VLP2000E	85 (21)		51 (17)		47 (28)	
Ciclo 12	Bolsa-válvula	97 (107)	0,9224	40 (51)	0,8654	47 (59)	0,8111
	VLP2000E	96 (22)		49 (18)		44 (28)	
Ciclo 14	Bolsa-válvula	105 (107)	0,9594	35 (35)	0,8477	38 (50)	0,9171
	VLP2000E	104 (20)		45 (16)		37 (24)	

Valores apresentados como média (desvio padrão); a: sangue arterial; P_CO2_: pressão parcial de dióxido de carbono; P_O2_: pressão parcial de oxigênio; S_O2_: saturação de oxigênio.

**Figura 2:**
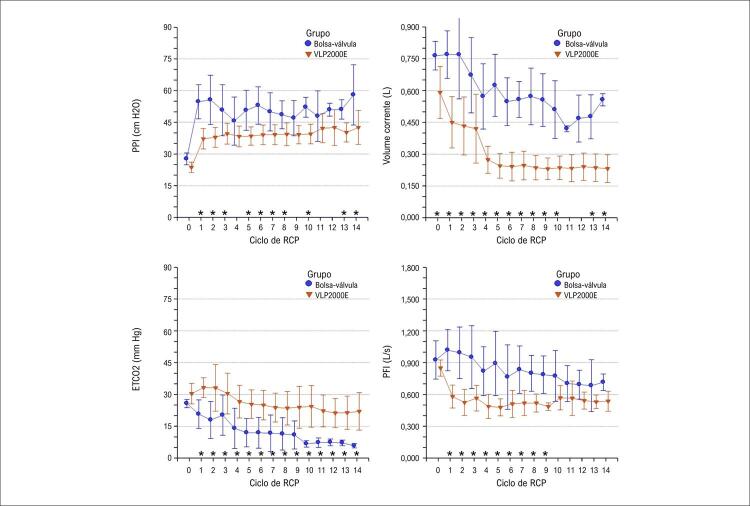
– Parâmetros respiratórios medidos antes da parada cardíaca no ciclo zero e nos 14 ciclos de ressuscitação cardiopulmonar (RCP), segundo o dispositivo de ventilação usado. Inicialmente com 10 porcos por grupo, do quarto ciclo de RCP ao 14º ciclo, o grupo bolsa-válvula incluiu quatro porcos e o grupo VLP2000E incluiu cinco porcos. Asteriscos indicam os ciclos com diferenças significantes; PPI: pico de pressão inspiratória; PFI: pico de fluxo inspiratório.

Os parâmetros respiratórios foram significantemente afetados pelo dispositivo de ventilação, como mostrado ciclo a ciclo na Figura 2 . Em 14 ciclos de RCP, a média de todas as medidas de cada parâmetro apresentou diferenças significantes (p < 0,0001) entre o grupo bolsa-válvula e o grupo VLP2000E no PPI [52 (9) vs. 39 (5) cm H_2_O], volume corrente [0,635 (0,172) vs. 0,306 (0,129) L], ETCO_2_ [14 (8) vs. 27 (9) mm Hg], e PFI [0,878 (0,234) vs. 0,533 (0,105) L/s], além do tempo inspiratório [1,96 (0,30) vs. 1,79 (0,18) s, p = 0,0154), respectivamente.

### Parâmetros após o RCE

**Figura 3:**
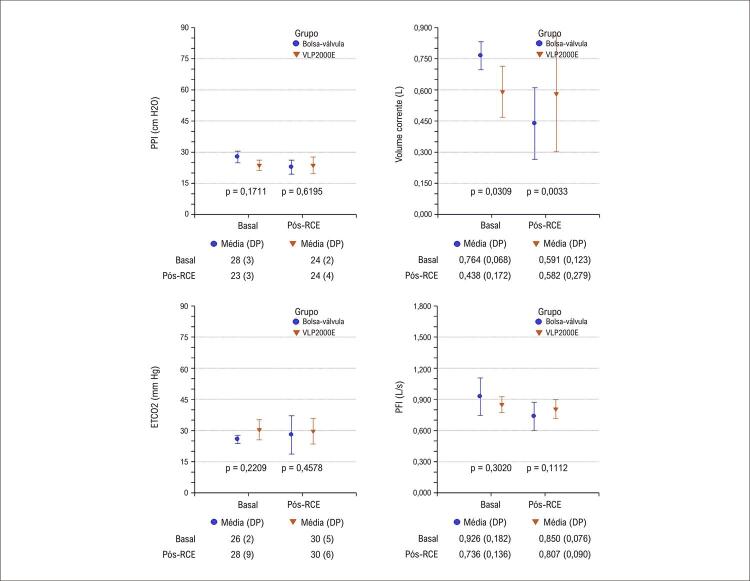
– Parâmetros respiratórios medidos antes da parada cardíaca (Basal) e durante alguns minutos de uso do dispositivo de ventilação usado na ressuscitação cardiopulmonar; pós-retorno da circulação espontânea (Pós-RCE), embora os parâmetros sejam apresentados por grupo, todos os animais foram ventilados com VLP2000E nesse período, cujos valores estatísticos correspondem a todas as medidas em cinco momentos; PPI: pico de pressão inspiratória; PFI: pico de fluxo inspiratório; DP: desvio padrão.

**Figura 4:**
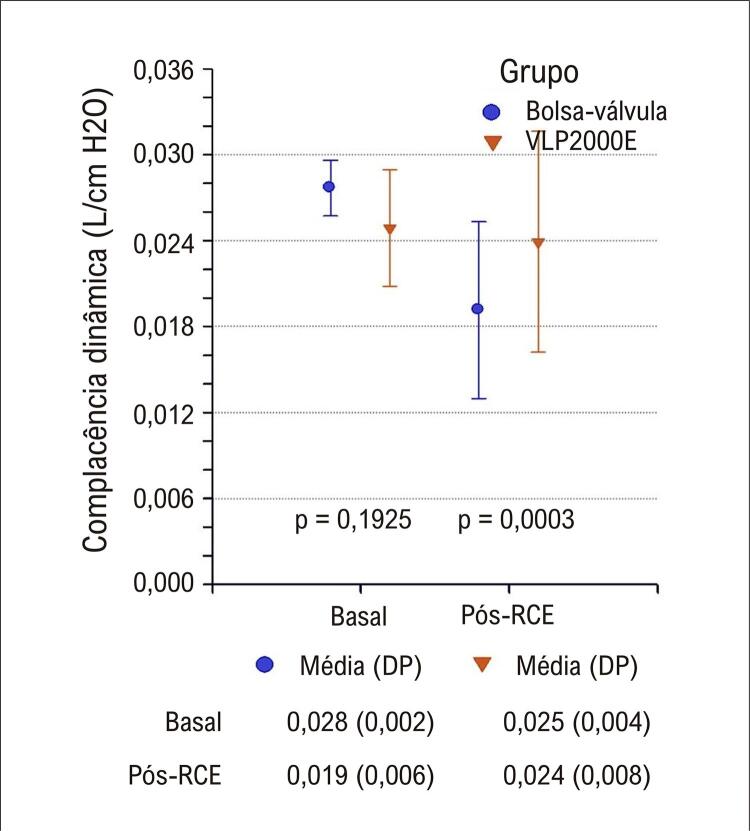
– Complacência dinâmica medida antes da parada cardíaca (Basal) e durante alguns minutos de uso do dispositivo de ventilação usado na ressuscitação cardiopulmonar; pós-retorno da circulação espontânea (Pós-RCE), embora os parâmetros sejam apresentados por grupo, todos os animais foram ventilados com VLP2000E nesse período, cujos valores estatísticos correspondem a todas as medidas em cinco momentos; PPI: pico de pressão inspiratória; PFI: pico de fluxo inspiratório; DP: desvio padrão.

Após o RCE, todos os animais foram ventilados com o VLP2000E; os valores dos parâmetros aos 10 minutos são apresentados na Tabela 3 . Nesse momento, observou-se diminuição significante da complacência dinâmica no grupo bolsa-válvula versus grupo VLP2000E [0,016 (0,006) vs. 0,022 (0,004) L/cm H_2_O, p = 0,0262]. Apesar do uso do mesmo ventilador e similares frequências respiratórias, níveis de fluxo e de pressão das vias aéreas após o RCE, os animais do grupo VLP2000E apresentaram valores maiores de volume corrente do que os animais ventilados com bolsa-válvula durante RCP ( Figura 3 ). A complacência pulmonar dinâmica era ≥ 0,025 L/cm H_2_O antes da parada cardíaca e, após o RCE, diminuiu significantemente após RCP com bolsa-válvula, mas não após RCP com VLP2000E ( Figura 4 ). Um resumo do estudo é apresentado na Figura Central .

**Tabela 3:** – Parâmetros medidos aos 10 minutos após o retorno da circulação espontânea, com todos os animais sendo ventilados com VLP2000E, segundo o dispositivo de ventilação usado na ressuscitação cardiopulmonar

PARÂMETRO	BOLSA-VÁLVULA	VLP2000E	p

	6 porcos	5 porcos	
P_aCO2_ (mm Hg)	37 (15)	41 (8)	0,7307
P_aO2_ (mm Hg)	190 (119)	265 (106)	0,0529
S_aO2_ (%)	94 (10)	98 (4)	0,6720
ETCO_2_ (mm Hg)	26 (14)	31 (9)	0,2004
Frequência respiratória (rpm)	19 (2)	19 (2)	0,9719
Frequência cardíaca (bpm)	89 (45)	128 (62)	0,0022
Débito cardíaco (L/min)	1,9 (0,3)	2,0 (0,3)	0,7483
Pressão aórtica (mm Hg)	57 (43)	60 (24)	0,8819
Pressão atrial direita (mm Hg)	17 (3)	11 (4)	0,0056
PIP (cm H_2_O)	21 (4)	19 (3)	0,7172
PIF (L/s)	0,628 (0,138)	0,729 (0,066)	0,2498
Volume corrente (L)	0,310 (0,096)	0,413 (0,091)	0,2387
Complacência dinâmica (L/cm H_2_0)	0,016 (0,006)	0,022 (0,004)	0,0262

Valores apresentados como média (desvio padrão); a: sangue arterial; P_CO2_: pressão parcial de dióxido de carbono; P_O2_: pressão parcial de oxigênio; S_O2_: saturação de oxigênio; ETCO_2_: dióxido de carbono expirado final; PPI: pico de pressão inspiratória; PFI: pico de fluxo inspiratório

**Figura Central:**
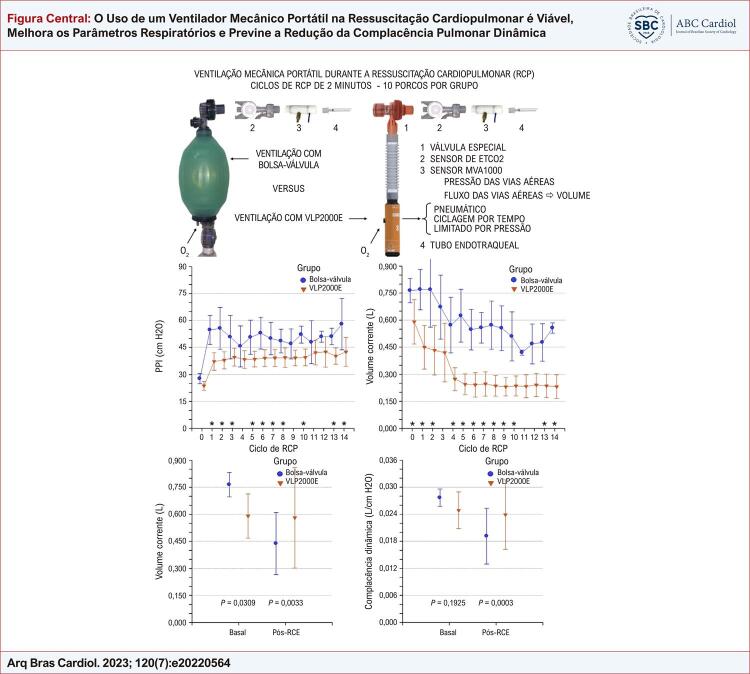
: O Uso de um Ventilador Mecânico Portátil na Ressuscitação Cardiopulmonar é Viável, Melhora os Parâmetros Respiratórios e Previne a Redução da Complacência Pulmonar Dinâmica

Um resumo do estudo.

## Discussão

A viabilidade do VLP2000E em ventilar simultaneamente com compressões torácicas foi avaliada neste estudo. Presumiu-se que a taxa de RCE seria menor no caso de ventilação insuficiente, e tal premissa foi usada para calcular o tamanho da amostra. Como observado, a taxa de RCE foi similar entre os dois grupos e comparável à taxa em outros estudos de RCP em porcos.^4 , 5^ Ainda, deve-se destacar a falta de diferenças significantes e relevantes nos parâmetros da gasometria, principalmente na saturação arterial de oxigênio. Recentemente, abordaram-se questões sobre ventilação mecânica durante RCP, sugerindo-se que o modo de ventilação pode não ser irrelevante na determinação dos resultados.^6^ Conforme a escolha dos ajustes no ventilador, no modo de ventilação pressão-controlada há o risco de não alcançar volume corrente suficiente, e no modo volume-controlado há o risco de exceder o nível seguro de PPI.^6^ Nossos resultados podem ajudar a responder essas questões, mostrando que o modo de ventilação pressão-controlada com pressão de 25 cm H_2_O (a máxima pressão inspiratória gerada pelo VLP2000E) foi suficiente quanto ao RCE e volume corrente alcançados, e a válvula especial foi eficaz em evitar nível elevado de PPI durante a ventilação com massagem cardíaca simultânea.

As diferenças significantes nos parâmetros respiratórios entre os grupos durante RCP e, após o RCE, na complacência pulmonar dinâmica, principalmente a manutenção da complacência demonstrada pela primeira vez neste estudo, sugerem que ventilação excessiva pode afetar os pulmões adversamente. Isso é consistente com outros estudos que não focaram predominantemente a parada cardíaca, e sim, a lesão pulmonar induzida por ventilador.^7 - 13^ Além da hiperventilação ligada à frequência respiratória elevada, o que se deveria evitar devido a efeitos hemodinâmicos deletérios, nosso estudo sugere que picos de pressão das vias aéreas e volumes correntes elevados também deveriam ser evitados devido a efeitos deletérios não só durante RCP como também após o RCE. O RCE foi alcançado após o máximo de três ciclos, e somente 4-6 minutos de RCP foram suficientes para reduzir a complacência dinâmica após ventilação com bolsa-válvula. Isso se deve, possivelmente, à elevada pressão nas vias aéreas (média de 52 cm H_2_O), considerando que já se descreveram modelos animais de lesão pulmonar aguda induzida por pressão tão alta quanto 50 cm H_2_O.^7 , 8^ Além da pressão elevada nas vias aéreas, observou-se grande volume corrente (média de 635 mL), com possível hiperdistensão regional, um importante fator relacionado a alterações que podem ter contribuído para a diminuição na complacência.^9 , 10^ Em porcos, observou-se complacência diminuída do sistema respiratório após quatro minutos de fibrilação ventricular e seis minutos de RCP e, em ratos, observou-se edema pulmonar causado por aumento de permeabilidade após cinco minutos de ventilação com pressões e volumes correntes elevados.^11 - 13^ Em contraste, após ventilação com VLP2000E com menor pressão (média de 39 cm H_2_O) e menor volume corrente (média de 306 mL), a complacência dinâmica permaneceu no nível basal.

Uma diminuição na complacência estática e dinâmica já foi observada após parada cardíaca e RCP, alertando que se deveria esperar lesão pulmonar na síndrome pós-parada cardíaca, o que pode ter impacto na morbidade e mortalidade.^11 , 14 , 15^ Sugere-se que essa diminuição na complacência esteja relacionada à congestão vascular, edema hidrostático, aspiração, e perda da capacidade residual funcional alveolar ou instabilidade alveolar.^14 - 20^ Tais considerações não explicam os resultados do nosso estudo, uma vez que a complacência dinâmica não diminuiu no grupo VLP2000E e, em ambos os grupos, a duração da parada cardíaca e da RCP, os níveis de PPC, e os valores de débito cardíaco após a ressuscitação foram similares. Por outro lado, alterações pulmonares relacionadas à ventilação excessiva podem explicar a diminuição na complacência dinâmica no grupo bolsa-válvula.^9 , 10 , 12 , 13 , 21^ Portanto, o efeito da compressão torácica adicionado ao PPI e ao volume corrente elevados podem levar à hiperdistensão pulmonar regional, diminuição no volume expiratório final e na capacidade residual funcional, combinado com a ausência de PEEP e possível redução no surfactante, predispõem à instabilidade alveolar. Alterações prováveis compostas de colapso alveolar e edema de permeabilidade podem explicar a diminuição na complacência no grupo bolsa-válvula.^9 , 10^

Aspectos prognósticos, fisiopatológicos e práticos importantes têm sido discutidos, relacionados à capnografia durante RCP.^1 , 22^ No entanto, inferências sobre o prognóstico ou qualidade das compressões torácicas de acordo com os níveis de ETCO_2_ podem ser inadvertidamente comprometidas por vários fatores de confusão, principalmente a falta de controle habitual e inconsistência durante RCP do produto entre frequência respiratória e volume corrente.^22^ Nosso estudo confirma a importância desse controle, mostrando o impacto das diferenças no produto sobre o ETCO_2_, uma vez que embora a frequência respiratória (assim como a PPC) não tenha diferido entre os grupos, o ETCO_2_ diferiu significantemente, com níveis inversamente proporcionais ao volume corrente. Recentemente, propôs-se a hipótese de oclusão das vias aéreas intratorácicas associada a volume pulmonar reduzido nos pacientes em parada cardíaca, o que poderia explicar a baixa ventilação alveolar e o comprometimento da troca gasosa durante RCP.^17 - 20^ A aplicação de um nível de PEEP adequado poderia prevenir a oclusão das vias aéreas sem um efeito hemodinâmico importante.^17 - 20^ Um índice de abertura das vias aéreas foi definido com base na curva de capnografia durante RCP, que reflete o grau de oscilação do sinal de CO_2_ acompanhando as descompressões torácicas.^18 , 20^ A oclusão das vias aéreas intratorácicas tende a ser mais intensa de acordo com a duração da parada cardíaca e da RCP, e a diminuição na complacência está relacionada a ambos os fatores.^16 , 18^ Em nosso estudo, uma vez que a duração da parada cardíaca e da RCP foi similar, e PEEP não foi aplicada em nenhum grupo, um maior grau de oclusão das vias aéreas intratorácicas levando à redução do ETCO_2_ e da complacência pulmonar somente no grupo bolsa-válvula é improvável.

### Aplicação clínica

O problema frequente de hiperventilação na RCP pode ser evitado com o uso do VLP2000E. O ventilador provê ainda ventilação automática, o que permite um menor número de socorristas, e o uso de um filtro de partículas aéreas com alta eficiência (filtro HEPA) entre o tubo endotraqueal (ou máscara facial) e a válvula especial reduz o risco de dispersão de partículas virais.^23^ Assim, esse ventilador pode ser apropriado para a segurança dos socorristas no tratamento de pacientes com parada cardíaca e suspeita ou confirmação de COVID-19.^23^ Essas qualidades podem tornar o VLP2000E uma opção notável como dispositivo de ventilação, principalmente fora do hospital, em unidades de transporte, doenças agudas, e RCP.

### Limitações

Os animais eram sadios antes do experimento, e esse modelo de RCP não simula casos de pacientes com doença pulmonar obstrutiva crônica ou asma. Não foram realizadas biópsia, necrópsia ou radiografia dos pulmões; tais medidas contribuiriam para o entendimento de causas potenciais da complacência pulmonar diminuída. Ainda, o experimento não foi delineado para observação tardia, a qual forneceria mais informações sobre a progressão da complacência dinâmica e os desfechos da parada cardíaca.

## Conclusões

Em um modelo suíno de parada cardíaca súbita e RCP, ventilação com VLP2000E é viável e equivalente a ventilação com bolsa-válvula quanto à taxa de RCE e saturação arterial de oxigênio. Esse ventilador produz melhores parâmetros respiratórios, com pressão das vias aéreas e volume corrente menores, mesmo durante ventilação e compressões torácicas simultâneas. Ventilação com VLP2000E também previne a redução significante da complacência pulmonar dinâmica observada após ventilação com bolsa-válvula. Seria interessante realizar mais estudos pré-clínicos para confirmar esses resultados.
