# Supplementary material for: O Uso de um Ventilador Mecânico Portátil na Ressuscitação Cardiopulmonar é Viável, Melhora os Parâmetros Respiratórios e Previne a Redução da Complacência Pulmonar Dinâmica
Source: Arq Bras Cardiol. 2023 Jul 19;120(7):e20220564. [Article in Portuguese] doi: 10.36660/abc.20220564 (PMC10382150; doi:10.36660/abc.20220564)
Supplement: Translation

# Translation: Use of a Portable Mechanical Ventilator during Cardiopulmonary Resuscitation is Feasible, Improves Respiratory Parameters, and Prevents the Decrease of Dynamic Lung Compliance

**Authors:** Manoel Ângelo Gomes Palácio, Edison Ferreira de Paiva, Gustavo Bernardes de Figueiredo Oliveira, Luciano César Pontes de Azevedo, Bruno Gregnanin Pedron, Elizabete Silva dos Santos, Ari Timerman

**Affiliations:** 1 Brazil Instituto Dante Pazzanese de Cardiologia, São Paulo, SP – Brazil; 2 Brazil Hospital Sírio-Libanês, São Paulo, SP – Brazil; 3 Brazil Universidade de São Paulo, São Paulo, SP – Brazil

**Keywords:** Hyperventilation, Lung Injury, Cardiopulmonary Resuscitation, Ventilators, Mechanical, Lung Compliance

## Abstract

**Background:**

For practical and protective ventilation during cardiopulmonary resuscitation (CPR), a 150-grams mechanical ventilator (VLP2000E) that limits peak inspiratory pressure (PIP) during simultaneous ventilation with chest compressions was developed.

**Objectives:**

To evaluate the feasibility of VLP2000E ventilation during CPR and to compare monitored parameters versus bag-valve ventilation.

**Methods:**

A randomized experimental study with 10 intubated pigs per group. After seven minutes of ventricular fibrillation, 2-minute CPR cycles were delivered. All animals were placed on VLP2000E after achieving return of spontaneous circulation (ROSC).

**Results:**

Bag-valve and VLP2000E groups had similar ROSC rate (60% vs. 50%, respectively) and arterial oxygen saturation in most CPR cycles, different baseline tidal volume [0.764 (0.068) vs. 0.591 (0.123) L, p = 0.0309, respectively] and, in 14 cycles, different PIP [52 (9) vs. 39 (5) cm H_2_O, respectively], tidal volume [0.635 (0.172) vs. 0.306 (0.129) L], ETCO_2_[14 (8) vs. 27 (9) mm Hg], and peak inspiratory flow [0.878 (0.234) vs. 0.533 (0.105) L/s], all p < 0.0001. Dynamic lung compliance (≥ 0.025 L/cm H_2_O) decreased after ROSC in bag-valve group but was maintained in VLP2000E group [0.019 (0.006) vs. 0.024 (0.008) L/cm H_2_O, p = 0.0003].

**Conclusions:**

VLP2000E ventilation during CPR is feasible and equivalent to bag-valve ventilation in ROSC rate and arterial oxygen saturation. It produces better respiratory parameters, with lower airway pressure and tidal volume. VLP2000E ventilation also prevents the significant decrease of dynamic lung compliance observed after bag-valve ventilation. Further preclinical studies confirming these findings would be interesting.

## Introduction

High-quality cardiopulmonary resuscitation (CPR) with adequate ventilation is currently prioritized, but hyperventilation remains a problem, even when CPR is delivered by well-trained teams.^1 , 2^ This calls for solutions such as the use of ventilation monitoring methods to guide ventilation delivery.^2^ In addition to causing deleterious hemodynamic effects, increased respiratory rates and tidal volumes during CPR can also damage the lungs.^1 , 2^ In critically ill patients on mechanical ventilation, precautions to avoid barotrauma, volutrauma, and atelectrauma are indicated.^3^ Thereby, decreased morbidity and mortality have been shown in cases of acute lung injury and respiratory distress syndrome with reductions in tidal volume and airway pressure.^3^

Aiming to achieve practical and protective ventilation during CPR, a portable mechanical ventilator that limits peak inspiratory pressure (PIP) was developed, the VLP2000E (Vent-Logos Ltda., Vitória, ES, Brazil). This study tested the VLP2000E in an animal model of sudden cardiac arrest. The objectives were to evaluate the feasibility of VLP2000E ventilation during CPR and to compare monitored parameters versus bag-valve ventilation.

## Methods

This was a randomized experimental study in a pig model of sudden cardiac arrest and current CPR designed to simulate a case of adult out-of-hospital cardiac arrest. Twenty pigs were randomly allocated 1:1 to bag-valve ventilation or to VLP2000E ventilation. The study was approved by the Institutional Animal Care and Use Committee (protocol number IACUC 2015-13 IEP-HSL).

### Details of the VLP2000E

The VLP2000E is a pneumatically powered, time-cycled, and pressure-limited mechanical ventilator weighing 150 grams. The device works automatically when connected to a portable oxygen tank, with easy adjustment of the respiratory rate. In addition, ventilation can also be manually triggered by pushing a button. It has mechanisms that allow spontaneous breathing, as well as a special valve that limits PIP even during simultaneous ventilation with chest compressions.

### Preparation

Female Landrace pigs weighing approximately 33 kg were fasted for 12 hours with free access to water prior to the procedure. After intramuscular anesthesia (5 mg/kg of ketamine hydrochloride [Ketalar 50 mg/mL, Pfizer]; 0.5 mg/kg of midazolam [Dormonid 5 mg/mL, Roche]), 12.5 mg/kg of thiopental (Thiopentax 20 mg/mL, Cristália) was infused through an ear vein, and the animal was intubated with a number seven endotracheal tube with cuff, which was inflated to avoid leaks. Continuous intravenous anesthesia was maintained with midazolam 1.5 mg/kg/h and 0.015 mg/kg/h of fentanyl citrate (Fentanyl 0.05 mg/mL, Janssen) plus 4-mL unrestricted boluses, and thiopental 0.6-6 mg/kg/h.

A Dräger Evita XL conventional mechanical ventilator (Drägerwerk AG & Co. KGaA, Lübeck, Germany) was used only during preparation, which was set to deliver intermittent positive pressure, tidal volume of 10 mL/kg, positive end-expiratory pressure (PEEP) of 5 cm H_2_O, and fraction of inspired oxygen (F_IO2_) of 50%; the respiratory rate was adjusted to maintain the end-tidal carbon dioxide (ETCO_2_) between 40-45 mm Hg.

The animals’ jugular veins and femoral arteries were punctured and cannulated with 8 French (F) and 6 F hemostatic introducers, respectively. The right jugular vein was used to infuse general anesthesia and all medications. A Swan-Ganz catheter of 7.5 F was placed with continuous cardiac output in the pulmonary artery via left jugular vein, and a pigtail catheter of 6 F was placed in the aortic root via right femoral artery.

### Monitoring, measured parameters, and blood sampling

The following equipment items were used: Dräger Infinity Delta XL multiparameter monitor (Dräger Medical Systems Inc., Telford, PA, USA); NICO ETCO_2_monitor (Koninklijke Philips Electronics NV, Amsterdam, Netherlands); Vigilance II cardiac output monitor (Edwards Lifesciences LLC, Irvine, CA, USA); MVA1000 airway monitor (Neurony Ltda., Vitória, ES, Brazil); Biopac MP100 data acquisition system with AcqKnowledge software (Biopac Systems Inc., Goleta, CA, USA); and Radiometer ABL735 blood gas analyzer (Radiometer Medical ApS, Brønshøj, Denmark).

The Biopac system recorded the following parameters at 250 samples per second: electrocardiogram (Biopac surface electrodes); aortic pressure (Biopac pressure transducer connected to pigtail catheter); right atrial pressure (Biopac transducer connected to Swan-Ganz atrial port); coronary perfusion pressure (CPP) (the difference between aortic and right atrial pressures); ETCO_2_(NICO analogue output connected to Biopac); cardiac output (Vigilance analogue output); and airway pressure and airway flow (MVA1000 analogue outputs). Tidal volume (integral of the airway flow curve at inspiration) and a simplified dynamic lung compliance (volume-pressure ratio at inspiration) were calculated. Central temperature was also monitored.

Blood samples were collected from left femoral artery (or from aorta during CPR) 30 minutes before the cardiac arrest (baseline), in CPR cycles 2, 4, 7, 9, 12, and 14, and after the return of spontaneous circulation (ROSC) at 10, 30, 60, 90, and 120 minutes. Blood gas and respiratory, and hemodynamic parameters (except for cardiac output, which is not usually measurable in CPR) were measured at these cycles and time points.

### Systematization of measurements

In all cycles and time points, three consecutive breaths were selected using AcqKnowledge software to measure each parameter and obtain the final mean of the respiratory and hemodynamic parameters. The following intervals were defined for measurement: the total respiratory cycle, the interval between two QRS complexes or two chest compressions, and the inspiration interval, providing the respiratory and heart rates, the chest compression rate, the PIP, the peak inspiratory flow (PIF), the tidal volume and the dynamic lung compliance. In the total respiratory cycle interval, ETCO_2_, aortic pressure, right atrial pressure, CPP, and cardiac output were measured.

### Experiment

Ventricular fibrillation was induced by applying direct current from a 9-V battery to the right ventricle for one second via a transvenous pacemaker electrode placed temporarily via right jugular vein. After seven minutes of inactivity to simulate the response time of emergency medical services, 2-minute CPR cycles (100 manual chest compressions per minute with approximately 5 cm deep and 10 breaths per minute) were initiated. Ten pigs were ventilated using an adult bag-valve with reservoir and oxygen influx at 10 L/min, and another 10 pigs were ventilated using the VLP2000E with F_IO2_of 100%. In both groups, PEEP was not applied, and oxygen was provided by a portable cylinder. Guided by a metronome, to avoid interpersonal bias, the same team member compressed the chest at a rate of 100 per minute; another member delivered 10 breaths per minute with bag-valve, delivering each breath in approximately two seconds following four beats of the metronome, and delivering the next breath on the 10^th^beat. The VLP2000E was adjusted to deliver 10 breaths per minute. After the first CPR cycle, defibrillation was attempted with 150 J, and CPR was resumed immediately. The cardiac rhythm was checked after each cycle, followed by another attempt with 150 J or CPR resumption. In cycles 2, 4, 7, 9, 12, and 14, adrenaline (Epinephrine 1 mg/mL, Cristália) 0.02 mg/kg was administered. In cycles 3 and 5, amiodarone (Ancoron 50 mg/mL, Libbs) 5 mg/kg and 2.5 mg/kg, respectively, were administered in cases of persistent ventricular fibrillation. CPR was continued for up to 30 minutes or until ROSC, and in cases of ROSC, all animals were placed on the VLP2000E for two hours before euthanasia with anesthetics plus 10 mL of potassium chloride. Sustained ROSC was indicated by an adequate cardiac rhythm and systolic pressure above 50 mm Hg for more than 10 minutes, and if spontaneous cardiac arrest occurred thereafter, the experiment was ended; however, if this occurred within less than 10 minutes, resuscitation was restarted until completing 30 minutes of CPR or until ROSC was achieved. Immediately after each ROSC episode in both groups, five vigorous consecutive breaths were delivered with bag-valve to expand the lungs equally in both groups.

### Statistical analysis

Adopting the assumption that resuscitation would not be impaired by mechanical ventilation, the sample size was calculated assuming a ROSC rate of 83%. Otherwise, if only 25% ROSC was achieved in the mechanical ventilation group, then 10 animals per group would be required for 80% statistical power and 5% two-tailed alpha.

The chi-square test or Fisher’s exact test, two-way ANOVA (time point, group), and Bonferroni multiple comparisons were performed using BioStat statistical software (AnalystSoft, Inc.). Graphs were generated with the Number Cruncher Statistical System (NCSS, LLC), and data are presented as mean (standard deviation) [mean (SD)]. Differences were considered significant at p < 0.05.

## Results

Preparation of the animals, whose mean body weight was 33 (2) kg, required approximately 90 minutes. During the experiment, the mean central temperature was 37.3 (1.3) °C. Following preparation and still with the conventional ventilator, the parameter values are shown in Table 1 . Figure 1 shows the bag-valve and the VLP2000E with accessories used in the experiment, in addition to schematically showing the position of respiratory sensors.

**Table 1:** – Parameters measured during conventional mechanical ventilation before cardiac arrest, according to the ventilation device used in cardiopulmonary resuscitation

PARAMETER	BAG-VALVE	VLP2000E	p

	10 pigs	10 pigs	
P_aCO2_(mm Hg)	44 (4)	43 (4)	0.8631
P_aO2_(mm Hg)	213 (19)	221 (16)	0.7822
S_aO2_(%)	99.9 (0.2)	99.9 (0.2)	0.9964
ETCO_2_(mm Hg)	43 (5)	42 (5)	0.7274
Respiratory rate (rpm)	16 (4)	16 (4)	0.6636
Heart rate (bpm)	74 (15)	62 (13)	0.2277
Cardiac output (L/min)	2.7 (0.7)	2.5 (0.6)	0.5523
Aortic pressure (mm Hg)	93 (21)	96 (17)	0.8107
Right atrial pressure (mm Hg)	13 (2)	12 (2)	0.7635

Values presented as mean (SD); a: arterial blood; P_CO2_: partial pressure of carbon dioxide; P_O2_: partial pressure of oxygen; S_O2_: oxygen saturation; ETCO_2_: end-tidal carbon dioxide.

**Figure 1:** – The bag-valve and the VLP2000E, and the positioning of respiratory sensors; CPR: cardiopulmonary resuscitation; ETCO2: end-tidal carbon dioxide.

### ROSC and 2-hour survival

Similar (p = 1.0000) ROSC rates were achieved with bag-valve ventilation (60%; three pigs after two CPR cycles, three pigs after three cycles) and VLP2000E ventilation (50%; two pigs after two CPR cycles, three pigs after three cycles). After 30 minutes of observation, three animals in the bag-valve group presented with spontaneous cardiac arrest. At 2 hours of observation, three pigs from the bag-valve group and five pigs from the VLP2000E group remained alive (p = 0.6499).

### Parameters monitored during ongoing CPR

Some parameters did not differ between the bag-valve and VLP2000E groups in all CPR cycles, including the chest compression rate [98 (2) vs. 99 (2) cpm, p = 0.8820], respiratory rate [11 (1) vs. 11 (1) rpm, p = 0.4477], and CPP, for instance in the cycle 1 [30 (17) vs. 35 (16) mm Hg, p = 0.6207], respectively. Blood gas parameters did not differ between the groups in most CPR cycles, except for initial relative hypercapnia in the VLP2000E group, as shown in Table 2 .

**Table 2:** – Arterial blood gas analyzed during cardiopulmonary resuscitation (CPR), according to the ventilation device used. Initially with 10 pigs per group, from the fourth CPR cycle to the 14th cycle, bag-valve group included four pigs, and VLP2000E group included five pigs

CPR	GROUP	P_aCO2_	p	P_aO2_	p	S_aO2_	p
		
		mm Hg		mm Hg		%	
Cycle 2	Bag-valve	27 (8)	0.0056	149 (63)	0.1040	97 (3)	0.0912
	VLP2000E	52 (13)		103 (90)		86 (13)	
Cycle 4	Bag-valve	35 (12)	0.0337	109 (99)	0.3025	79 (24)	0.3232
	VLP2000E	64 (19)		65 (20)		69 (22)	
Cycle 7	Bag-valve	29 (2)	0.0111	83 (24)	0.6193	90 (7)	0.0105
	VLP2000E	72 (20)		56 (13)		58 (21)	
Cycle 9	Bag-valve	94 (92)	0.6098	41 (44)	0.8552	45 (59)	0.8714
	VLP2000E	85 (21)		51 (17)		47 (28)	
Cycle 12	Bag-valve	97 (107)	0.9224	40 (51)	0.8654	47 (59)	0.8111
	VLP2000E	96 (22)		49 (18)		44 (28)	
Cycle 14	Bag-valve	105 (107)	0.9594	35 (35)	0.8477	38 (50)	0.9171
	VLP2000E	104 (20)		45 (16)		37 (24)	

Values presented as mean (SD); a: arterial blood; P_CO2_: partial pressure of carbon dioxide; P_O2_: partial pressure of oxygen; S_O2_: oxygen saturation.

Respiratory parameters were significantly affected by the ventilation device, as shown on a cycle-by-cycle basis in Figure 2 . In 14 CPR cycles, the mean of all measurements of each parameter showed significant differences (all p < 0.0001) between bag-valve group and VLP2000E group in PIP [52 (9) vs. 39 (5) cm H_2_O], tidal volume [0.635 (0.172) *vs.* 0.306 (0.129) L], ETCO_2_[14 (8) *vs.* 27 (9) mm Hg], and PIF [0.878 (0.234) *vs.* 0.533 (0.105) L/s], in addition to inspiratory time [1.96 (0.30) *vs.* 1.79 (0.18) s, p = 0.0154), respectively.

**Figure 2:** – Respiratory parameters measured before cardiac arrest at cycle zero and in 14 CPR cycles, according to the ventilation device used. Initially with 10 pigs per group, from the fourth cycle to the 14th cycle, bag-valve group included four pigs, and VLP2000E group included five pigs. Asterisks indicate cycles with significant differences; PIP: peak inspiratory pressure; PIF: peak inspiratory flow.

### Parameters after ROSC

After ROSC, all animals were placed on the VLP2000E; the parameter values at 10 minutes are shown in Table 3 . At this time point, a significant decrease of dynamic compliance was observed in the bag-valve group versus the VLP2000E group [0.016 (0.006) vs. 0.022 (0.004) L/cm H_2_O, p = 0.0262]. Despite the use of the same ventilator and similar respiratory rates, flow levels, and airway pressures after ROSC, the animals in the VLP2000E group had higher tidal volume than the animals ventilated with bag-valve during CPR, as shown in Figure 3 . Dynamic lung compliance was ≥ 0.025 L/cm H_2_O prior to cardiac arrest and, following ROSC, it decreased significantly after CPR with bag-valve ventilation, but not after CPR with VLP2000E ventilation ( Figure 4 ). A summary of the study is shown in Central Illustration .

**Table 3:** – Parameters measured at 10 minutes after return of spontaneous circulation with all animals placed on the VLP2000E, according to the ventilation device used in cardiopulmonary resuscitation

PARAMETER	BAG-VALVE	VLP2000E	p

	6 pigs	5 pigs	
P_aCO2_(mm Hg)	37 (15)	41 (8)	0.7307
P_aO2_(mm Hg)	190 (119)	265 (106)	0.0529
S_aO2_(%)	94 (10)	98 (4)	0.6720
ETCO_2_(mm Hg)	26 (14)	31 (9)	0.2004
Respiratory rate (rpm)	19 (2)	19 (2)	0.9719
Heart rate (bpm)	89 (45)	128 (62)	0.0022
Cardiac output (L/min)	1.9 (0.3)	2.0 (0.3)	0.7483
Aortic pressure (mm Hg)	57 (43)	60 (24)	0.8819
Right atrial pressure (mm Hg)	17 (3)	11 (4)	0.0056
PIP (cm H_2_O)	21 (4)	19 (3)	0.7172
PIF (L/s)	0.628 (0.138)	0.729 (0.066)	0.2498
Tidal volume (L)	0.310 (0.096)	0.413 (0.091)	0.2387
DCompliance (L/cm H_2_0)	0.016 (0.006)	0.022 (0.004)	0.0262

Values presented as mean (SD); a: arterial blood; P_CO2_: partial pressure of carbon dioxide; P_O2_: partial pressure of oxygen; S_O2_: oxygen saturation; ETCO_2_: end-tidal carbon dioxide; PIP: peak inspiratory pressure; PIF: peak inspiratory flow; DCompliance: dynamic compliance.

**Figure 3:** – Respiratory parameters measured before cardiac arrest (Baseline) during a few minutes’ use of the ventilation device used in cardiopulmonary resuscitation; post-return of spontaneous circulation (Post-ROSC), the parameters are presented according to the groups, but all animals were placed on the VLP2000E in this period, in which the statistics correspond to all measurements in five time points; PIP: peak inspiratory pressure; PIF: peak inspiratory flow.

**Figure 4:** – Dynamic compliance measured before cardiac arrest (Baseline) and during a few minutes’ use of the ventilation device used in cardiopulmonary resuscitation; post-return of spontaneous circulation (Post-ROSC), compliance is shown according to the groups, but all animals were placed on the VLP2000E in this period, in which the statistics correspond to all measurements in five time points.

**Central Illustration:** : Use of a Portable Mechanical Ventilator during Cardiopulmonary Resuscitation is Feasible, Improves Respiratory Parameters, and Prevents the Decrease of Dynamic Lung Compliance

A summary of the study.

## Discussion

The feasibility of the VLP2000E to deliver ventilation simultaneously with chest compressions was evaluated. The ROSC rate was assumed to be lower in cases of insufficient ventilation, and this assumption was used to calculate the sample size. As observed, ROSC rate was similar between the two groups and comparable to the rate in other studies of CPR in pigs.^4 , 5^ In addition, the lack of relevant and significant differences in blood gas parameters must be noted, especially those in the arterial oxygen saturation. Recently, questions about mechanical ventilation during CPR were addressed, noting that the ventilatory mode may not be irrelevant in determining results.^6^ According to the choice of ventilator settings, in pressure-controlled mode, there is a risk of not reaching sufficient tidal volume, and in volume-controlled mode, there is a risk of exceeding the safe PIP level.^6^ Our results may help answer these questions by showing that the pressure-controlled ventilation mode with a pressure of 25 cm H_2_O (the maximum inspiratory pressure generated by the VLP2000E) was sufficient regarding the ROSC and tidal volume achieved, and the special valve was effective in avoiding high PIP level during ventilation with concomitant chest compressions.

The significant differences in respiratory parameters between groups during CPR and, after ROSC, in dynamic lung compliance, especially the maintenance of compliance demonstrated for the first time by this study, suggest that excessive ventilation may adversely affect the lungs. This is consistent with other studies that did not focus on cardiac arrest but, instead, on ventilator-induced lung injury.^7 - 13^ In addition to hyperventilation related to high respiratory rate, which should be avoided due to deleterious hemodynamic effects, our study suggests that high airway pressure peaks and tidal volumes should also be avoided due to deleterious effects not only during CPR but also after ROSC. The ROSC was achieved after three cycles at most, and only 4-6 minutes of CPR were sufficient to reduce dynamic compliance after bag-valve ventilation, which may have been due to the high airway pressure (mean of 52 cm H_2_O), considering that animal models of airway pressure-induced acute lung injury at pressures as high as 50 cm H_2_O have been described.^7 , 8^ Along with high airway pressure, high tidal volume (mean of 635 mL) was observed, with possible regional overdistention, an important factor related to alterations that may have contributed to the decrease in compliance.^9 , 10^ In pigs, decreased respiratory system compliance after four minutes of ventricular fibrillation and six minutes of CPR have been observed, while in rats, pulmonary oedema has been observed due to increased permeability after five minutes of ventilation with high pressures and high tidal volumes.^11 - 13^ In contrast, after VLP2000E ventilation with lower airway pressure (mean of 39 cm H_2_O) and tidal volume (mean of 306 mL), dynamic compliance remained at baseline level.

A decrease in static and dynamic compliance has been observed after cardiac arrest and CPR, warning that lung injury should be expected in post-cardiac arrest syndrome, which may have an impact on morbidity and mortality.^11 , 14 , 15^ This decrease in compliance has been suggested to be related to vascular congestion, hydrostatic oedema, aspiration, and loss of alveolar functional residual capacity or alveolar instability.^14 - 20^ These considerations do not explain the results of our study because dynamic compliance did not decrease in the VLP2000E group and, in both groups, the duration of cardiac arrest and CPR, CPP levels, and post-resuscitation cardiac output levels were similar. In contrast, pulmonary alterations related to excessive ventilation may explain the decrease in dynamic compliance in the bag-valve group.^9 , 10 , 12 , 13 , 21^ Therefore, the effect of chest compression added to high PIP and tidal volume may lead to regional lung overdistention, decrease in final expiratory volume and functional residual capacity, combined with the absence of PEEP and possible decrease in surfactant predispose to alveolar instability. Probable alterations composed of alveolar collapse and permeability oedema may explain the decrease in compliance in the bag-valve group.^9 , 10^

Important practical, pathophysiological, and prognostic aspects related to capnography during CPR have been discussed.^1 , 22^ However, inferences regarding the prognosis or quality of chest compressions according to ETCO_2_levels may be inadvertently compromised by several confounding factors, especially the lack of usual control and inconsistency during CPR of the product of respiratory rate and tidal volume.^22^ Our study confirms the importance of this control, showing the impact of differences in the product on ETCO_2_, since respiratory rate (like CPP) did not differ between groups during CPR, but the ETCO_2_differed significantly, with level inversely proportional to the tidal volume. Recently, the hypothesis of intrathoracic airway closure associated with reduced lung volume in patients with cardiac arrest has been proposed, which may explain the low alveolar ventilation and gas exchange impairment during CPR.^17 - 20^ Applying an adequate PEEP level can prevent airway closure without significant hemodynamic effect.^17 - 20^ An airway opening index has been defined based on waveform capnography during CPR, which reflects the degree of oscillation of the CO_2_signal accompanying chest decompressions.^18 , 20^ Intrathoracic airway closure tends to be more intense according to the duration of cardiac arrest and CPR, and the decrease in compliance is related to both factors.^16 , 18^ In our study, because the duration of cardiac arrest and CPR was similar, and PEEP was not applied to any group, a greater degree of intrathoracic airway closure decreasing ETCO_2_and lung compliance only in the bag-valve group is unlikely.

### Clinical application

The frequent problem of hyperventilation in CPR can be avoided with the use of the VLP2000E. It also provides automatic ventilation, which allows a reduction in the number of rescuers, and the use of a high-efficiency particulate air (HEPA) filter between the endotracheal tube (or face mask) and the special valve reduces the risk of aerosolization of viral particles.^23^ Therefore, this ventilator may be appropriate for the safety of rescuers when treating patients with cardiac arrest with suspected or confirmed COVID-19.^23^ These qualities may render the VLP2000E a noteworthy option as ventilation device, especially in the field, transport units, acute diseases, and CPR.

### Limitations

The animals were healthy prior to the experiment, and this model of CPR does not simulate cases of patients with chronic obstructive pulmonary disease or asthma. Lung biopsy or necropsy specimens were not analyzed, nor were the lungs X-rayed; such measures may contribute to the understanding of potential causes of decreased lung compliance. In addition, the experiment was not planned for late observation, which could provide more information on the progression of dynamic compliance and on cardiac arrest outcomes.

## Conclusions

In a pig model of sudden cardiac arrest and CPR, VLP2000E ventilation is feasible and equivalent to bag-valve ventilation in ROSC rate and arterial oxygen saturation. It produces better respiratory parameters, with lower airway pressure and tidal volume, even during simultaneous ventilation with chest compressions. VLP2000E ventilation also prevents the significant decrease of dynamic lung compliance observed after bag-valve ventilation. Further preclinical studies confirming these findings would be interesting.
